# Exploring the Impact of Target Eccentricity and Task Difficulty on Covert Visual Spatial Attention and Its Implications for Brain Computer Interfacing

**DOI:** 10.1371/journal.pone.0080489

**Published:** 2013-12-03

**Authors:** Linsey Roijendijk, Jason Farquhar, Marcel van Gerven, Ole Jensen, Stan Gielen

**Affiliations:** Radboud University Nijmegen, Donders Institute for Brain, Cognition and Behaviour, Nijmegen, The Netherlands; Barrow Neurological Institute, United States of America

## Abstract

**Objective:**

Covert visual spatial attention is a relatively new task used in brain computer interfaces (BCIs) and little is known about the characteristics which may affect performance in BCI tasks. We investigated whether eccentricity and task difficulty affect alpha lateralization and BCI performance.

**Approach:**

We conducted a magnetoencephalography study with 14 participants who performed a covert orientation discrimination task at an easy or difficult stimulus contrast at either a near (3.5°) or far (7°) eccentricity. Task difficulty was manipulated block wise and subjects were aware of the difficulty level of each block.

**Main Results:**

Grand average analyses revealed a significantly larger hemispheric lateralization of posterior alpha power in the difficult condition than in the easy condition, while surprisingly no difference was found for eccentricity. The difference between task difficulty levels was significant in the interval between 1.85 s and 2.25 s after cue onset and originated from a stronger decrease in the contralateral hemisphere. No significant effect of eccentricity was found. Additionally, single-trial classification analysis revealed a higher classification rate in the difficult (65.9%) than in the easy task condition (61.1%). No effect of eccentricity was found in classification rate.

**Significance:**

Our results indicate that manipulating the difficulty of a task gives rise to variations in alpha lateralization and that using a more difficult task improves covert visual spatial attention BCI performance. The variations in the alpha lateralization could be caused by different factors such as an increased mental effort or a higher visual attentional demand. Further research is necessary to discriminate between them. We did not discover any effect of eccentricity in contrast to results of previous research.

## Introduction

Brain computer interfaces (BCIs) are systems that allow individuals to control a device by performing a mental task. The basic idea of BCIs is that different mental tasks, such as selective attention to sensory perception or motor imagery, cause different patterns of brain activity [1].These patterns can be measured and might be translated into various commands for a device, for example a computer or a wheelchair. An important aim of BCI is to facilitate the communication of patients with severe motor disabilities, such as amyotrophic lateral sclerosis (ALS), spinal cord injury, stroke and cerebral palsy [1]–[3].

A relatively new mental task for BCI is covert visual spatial attention, in which people visually attend to eccentric stimuli without moving their eyes [4]–[7]. Typically, subjects focus their eyes on a central fixation point on the screen, while attending to a target in the left or right visual field. During this covert attention task, differences in patterns of oscillatory brain activity or the BOLD signal are used for BCI control.

During covert visual spatial attention to the left or right visual field a typical alpha power lateralization (8–14 Hz) in the posterior cortex is observed in electrophysiological brain activity (see [8] for a review on lateralized alpha oscillations). During the attention period, posterior alpha oscillations desynchronize in the contralateral hemisphere and synchronize in the ipsilateral hemisphere [9], [10]. The alpha lateralization index, the ratio between left and right posterior alpha power, can then be used to control a BCI [11]. Not only left versus right attention can be detected in the brain signals; Bahramisharif et al. [12] demonstrated that it is even possible to decode the direction of covert attention to a target rotating along a circular trajectory with a mean absolute deviation of 70°. Treder et al. [13] also manipulated the direction to which subjects had to attend (for example upper left versus lower right visual field attention) and showed that subjects had different opposite pairs of directions in which they performed best.

As covert visual spatial attention is a relatively new BCI paradigm it is important to investigate which experimental parameters can possibly improve the BCI performance. Bahramisharif et al. [14] sought to improve the performance of the covert visual spatial attention BCI by modulating the eccentricity of the target. In their experiment, subjects had to covertly attend to stimuli at an eccentricity of 3°, 6°, or 9° from the central fixation point in the left and right hemifield. The results showed that the alpha lateralization pattern became more pronounced when the target eccentricity increased and that a minimum of 6° was necessary for sufficiently accurate classification of left versus right spatial attention. Indications of a retinotopical organization [15] were also observed in the lateralization pattern. In other words, spatial attention to a specific location could cause stronger activation in specific parts of the visual cortex.

However, a possible confound in the study of Bahramisharif et al. [14] was task difficulty. In Bahramisharif et al's study the cortical magnification factor [16] was not taken into account which could make the covert attention task easier for targets near the fovea than for more eccentric targets, since targets near the fovea activate a much larger cortical volume than equally-sized targets at more eccentric retinal locations.

Previous research has already demonstrated effects of task difficulty on behavioural performance [17] and on the neuronal firing rate in the visual cortex of monkeys [18], [19]. However, no study has shown an effect of task difficulty on activity in the alpha band in a covert visual spatial attention task to our knowledge. An effect is expected as it is known that task difficulty manipulates mental effort and mental effort influences alpha band activity [20], [21]. Passive BCIs that use the visual alpha band activity for mental workload detection have already been developed [22]. Yet, the effect of task difficulty on visual alpha lateralization, which is used as control signal in active BCIs, has not been investigated before.

The aim of our study was to explore how two possible properties, eccentricity and task difficulty, influence the power of alpha oscillations in a covert visual spatial attention task. We specifically investigated the magnitude of alpha lateralization in the visual cortex whereby we manipulated the two properties with a two by two design with factors Eccentricity (near, far) and Task Difficulty (easy, difficult). Furthermore, we investigated which of these conditions might give the best BCI performance.

## Materials and Methods

We set up a left versus right visual field covert spatial attention magnetoencephalography (MEG) experiment with an orientation discrimination task in which we manipulated task difficulty by using a low or high-contrast target stimulus and manipulated eccentricity with two target locations at either 3.5° or 7°.

### Ethics Statement

The procedures used in the experiment were according the Declaration of Helsinki, and all subjects gave written informed consent. The procedures were approved by the local ethics committee (Committee on- Research Involving Human Subjects, Region Arnhem-Nijmegen, The Netherlands).

### Subjects

Twenty-two healthy participants took part in the experiment. All participants had normal or corrected-to-normal vision and were right-handed. None of the subjects had any known neurological or psychiatric disorder. Participants gave written consent prior to the start of the experiment and were paid in accordance with the guidelines of the local ethics committee. Five participants were excluded from further analysis due to excessive eye movements to eccentric locations in more than a fourth of the trials during the experiment, which we interpreted as violation of the concept of covert attention to the target locations. Two other participants were excluded because of chance level performance in the discrimination task in the easy condition and one participant was excluded because of excessive head movements, which added artefacts to the MEG data. Therefore, a total of 14 subjects (mean age 24.8, range 19 37 years; 8 female) was taken into account for analysis. Three of these subjects had experience with covert visual spatial attention from a previous experiment.

### Data acquisition

A whole-head MEG system with 275 axial gradiometers (CTF MEG Systems, Port Coquitlam, Canada) was used to record ongoing brain activity at a sampling rate of 1200 Hz. The subject's head location relative to the MEG sensors was measured with marker coils placed at the nasion, and in the left and right ear canals. Sensors ‘MLT37’ and ‘MLF62’ could not be recorded for the first eleven subjects and sensors ‘MRF66’ and ‘MRO52’ could not be recorded for the last five subjects because of sensor dropout.

An EyeLink 1000 eye-tracker (SR Research Ltd., Kanata, Canada) pointing at the subject's right eye was used to measure eye movements with a sampling rate of 2000 Hz. At the beginning of each experiment subjects performed a calibration block to determine the gaze direction when overtly fixating. In this calibration procedure filled circles with a diameter of 1.04° were randomly shown for one second on the horizontal and vertical axes of the fixation cross at eccentricities of 3.5°, 7°, and 9°. The average calibration error was 0.2°.

### Stimulus presentation

Subjects were seated upright in the MEG system with their eyes 85 cm from a screen on which the stimuli were projected. The projection screen had a width of 45 cm. Stimuli were projected on the screen with an EIKI LC-XL100 projection system with a resolution of 1024×768 pixels, and a frame rate of 60 Hz. Stimuli were presented using Psychtoolbox (https://psychtoolbox.org). Gamma correction (luminance calibration) was applied to increase the brightness and contrast of the display.

### Experimental paradigm

**Figure 1:**
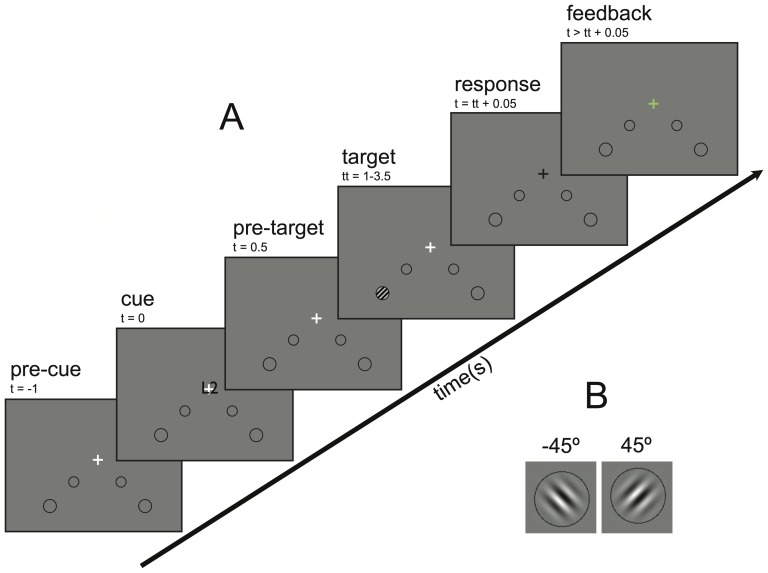
Schematic representation of the experimental design. (A) Trial timeline. Each trial started with a 1 second baseline period (pre-cue period), after which a letter cue was shown for 0.5 s (‘L1’ = near-left and ‘R1’ = near-right at 3.5° eccentricity; ‘L2’ = far-left and ‘R2’ = far-right at 7° eccentricity). Next, subjects had to covertly attend to the placeholder at the cued location whilst fixating at the white fixation cross for a random period in the range between 0.5 and 3 seconds (pre-target period). Then, a target was shown within the cued placeholder for 0.05 s. Next, the fixation cross turned from white to black and the subject had to press a button to indicate which target (−45° or +45°) he/she detected. Finally, feedback was shown (green cross = correct, red cross = incorrect) and after two seconds the next trial started. (B) Targets. Gabor patches with left orientation (−45°) or right orientation (+45°), shown here at full contrast.

Subjects were asked to perform a covert visual spatial attention task in which they had to direct their attention to one out of four different locations on the screen (near-left, near-right, far-left, far-right; see Figure 1A), while maintaining gaze at a fixation cross at the centre of the screen. At the attended location they had to perform a two-alternative forced choice Gabor patch orientation discrimination task at one of 2 difficulty levels; ‘easy’ and ‘difficult’. In the easy condition the Gabor patch had a full contrast (1∶1000) such that subjects performed the discrimination task approximately 100% correct, while in the difficult condition the patch contrast was set for each subject individually such that each subject performed the discrimination task approximately 75% correct. The contrast of the patch, defined as 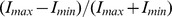, was 0.048, 0.370, 0.125, 0.250, 0.083, 0.104, 0.119, 0.175, 0.106, 0.120, 0.083, 0.094, 0.125, 0.087 for our 14 subjects, respectively.

### Stimuli

Stimuli were carefully chosen to maximize effects of eccentricity and task difficulty. Subjects attended to four spatial locations on a grey background at either 3.5° (near) or 7° (far) eccentricity at the lower left or lower right of the fixation cross (illustrated in Figure 1A). To help subjects maintain their attention at one of these locations black circles were used as placeholders. All four placeholders were shown during the entire experiment to minimize stimulus related effects. Attention was directed to the lower visual field because it has been shown that the resolution of spatial attention is better in the lower visual field than in the upper visual field [23].

The eccentricities of 3.5° and 7° and the sizes of the placeholders (1.04°, and 1.40° for the near and far location, respectively) were chosen for several reasons. Firstly, subjects should attend to visual targets outside their fovea as is standard in the covert visual attention paradigm. Secondly, the distance between the placeholders should be large enough to activate as much as possible disjunct regions in the visual cortex. Thirdly, the area within the placeholders should be such that each stimulus activates approximately equally-sized populations of neurons in the visual cortex involved in processing. The relation between the cortical magnification factor M and the retinal eccentricity E was provided by Duncan and Boyton [16] by the formula 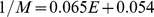. With eccentricities of 3.5° and 7° we ensured a distance of about 1 cm between the centres of gravity of the two populations of neurons in the primary visual cortex related to the near and far stimulus [16]. Furthermore, we corrected the diameters of the placeholders by the cortical magnification factor to ensure that the neuronal populations in visual cortex that are involved in visual processing were equally large, leading to diameters of 1.04° for near placeholders and 1.40° for far placeholders.

As targets, black-and-white Gabor patches were used that were constructed using four cycle sinusoidal waves with a 2-D Gaussian mask of 52% of the placeholder's width. Thus, near and far targets had the same number of waves. The sinusoid was oriented either at −45° (left) or +45° (right) as shown in Figure 1B.

#### Trial design

A trial (illustrated in Figure 1A) started with a white fixation cross (horizontal and vertical bar of 1° each) in the centre of the screen for 1 second, signalling the subject to start attending to the location of the cross (pre-cue period). Next, a letter cue was shown over the fixation cross for 0.5 second, indicating the location that had to be attended: ‘L1’ (near-left), ‘L2’ (far-left), ‘R1’ (near-right), and ‘R2’ (far-right). We used symbol codes in the centre of the screen instead of an indication at the location to be attended to prevent subjects from already exogenously attending to the target. After a period randomly chosen in the range 0.5–3 s (pre-target period), a target (a Gabor patch) was shown within the attended placeholder for three screen frames (0.05 s), directly followed by the fixation cross turning from white to black to inform subjects that the patch was shown. Ninety percent of the pre-target periods were generated according to a decaying exponential distribution in the range from 2 to 3 s with a mean of 2.5 s to prevent subjects from expecting a target at a specific moment in time. Ten percent of the pre-target periods were generated according to an exponential distribution in the range from 0.5 to 2 s with a mean of 0.8 s to ensure that subjects started attending immediately after cue onset.

Subjects were instructed to respond as accurately and as rapidly as possible to the orientation of the Gabor patch. They were required to press a button with their left index finger when the grating had an orientation of −45° and with their right middle finger when the grating had an orientation of +45°. After the response, feedback was given to the subject about the correctness of the response. The colour of the fixation cross turned green for a correct response and red for an incorrect response. Subsequently, after two seconds, the next trial began.

#### Block design

Each experiment began with a short practice period of 26 trials to familiarize the subject with the task. Next, subjects performed two blocks (88 trials in total) with varying target contrast to determine the target contrast for the difficult blocks. We used an adaptive method (Quest; [24]) to estimate the contrast level at which a subject performed the task approximately 75% correct. In the easy blocks the Gabor patch had full contrast (1∶1000) such that subjects performed the discrimination task approximately 100% correct. Finally, subjects performed five easy and five difficult blocks in pseudo-randomized order (440 trials total). The first two blocks consisted of an easy and difficult block in random order to allow the subject to experience both types of blocks early in the experiment. The direction of attention was pseudo-randomized and equally distributed (50% left, 50% right) in every block. The presentation of the grating orientation was also randomized (50% left, 50% right). After every two blocks subjects were forced to rest for one minute with their eyes closed and after every 22 trials they were allowed a short rest. They could indicate the time to continue by pressing a button.

### Data analysis

Data were analysed using FieldTrip, an open-source analysis Matlab toolbox for analysing electrophysiological data [25]. For classification of left versus right spatial attention data the Donders Machine Learning toolbox was used (https://github.com/distrep/DMLT). Next, SPSS was used for statistical analyses. Only trials with a minimum pre-target period of two seconds were used for analyses, such that all trials used contained a period of 1.5 seconds of covert attention without possible cue-related artefacts.

#### Behaviour

Behavioural performance was assessed by the percentage of correct responses on the discrimination task (behavioural accuracy) and the corresponding response times (RTs). Trials in which subjects responded very rapidly (<0.3 s) or slowly (>2 s) were excluded from the RT analysis. To evaluate the effects of eccentricity and task difficulty on the behavioural accuracy and response times, two-way repeated measures ANOVA with factors Eccentricity (near, far) and Task Difficulty (easy, difficult) were applied. Furthermore, to investigate the relation between the accuracy and the response times within subjects, a one-way repeated measures ANOVA with factor Interval was used. Interval was defined as a four-level factor in which each level indicated the accuracy in subject-specific time intervals where each interval contained 25% of the subject's trials.

#### MEG preprocessing

The acquired MEG data (sampled at 1200 Hz) was down-sampled to 300 Hz and data segments were defined as the period from one second before cue presentation until one second after target presentation. These data segments were demeaned and thereafter the data segments were cleaned in several steps. First, segments from trials in which subjects responded very rapidly (<0.3 s) or slowly (>2 s) were removed, which was the case for 0.6% and 0.3% of the trials respectively. In addition, trials in which fixation deviated more than two degrees away from the fixation cross for a period exceeding 0.05 s in the period between 1 s and 2.5 s after cue onset were removed (7.3% of trials). To correct for drift, the mean gaze position in each 1-second pre-cue period was used as a baseline for fixation. Next, trials in which the power averaged over the period between 1 s and 2.5 s after cue onset deviated more than three standard deviations from the average power in this period over all trials were removed, hereby removing possible EMG artefacts, MEG sensor jumps, or other noise (1.4% of trials). Data, which was collected with our MEG system with axial gradiometers, were transferred to synthetic planar gradient data to obtain the strongest activity directly above the sources [26]. The planar gradient was approximated for each sensor using the signals calculated from a sensor and its neighbouring sensors.

#### Time-frequency analysis

We used a Fourier transform combined with a Hanning taper method [27] to calculate the power in the alpha frequency range (8 to 14 Hz with 2 Hz bins) for the time interval between one second before cue onset until one second after target onset for each trial and each sensor separately. We used a 0.5 s sliding time window that was advanced in steps of 0.05 s. For each data segment, we averaged the Fourier transformed data over all frequency bins in the range from 8 to 14 Hz. This resulted in 273 signals (the number of recorded MEG sensors), representing the average power in the range between 8 and14 Hz in each sensor as a function of time. This will be referred to as the alpha power for all sensors. These data were used to analyse the data for three purposes: alpha power analysis (without time resolution), alpha time-power analysis (with time resolution), and classification analysis for BCI purposes.

#### Alpha power analysis

To investigate which sensors revealed the largest variations in alpha power in the covert visual spatial attention task, we averaged the alpha power for all sensors over the period from 1 s until 2.5 s after cue onset, resulting in a vector with 273 components for each trial. The period from 1 s until 2.5 s after cue onset was chosen because all data segments had a minimum period of 2.5 seconds after cue onset and the first second may contain cue-related information, which we wanted to exclude from our analysis. Next, we averaged over trials for each combination of subject, attended hemifield (left, right), and condition (easy near, easy far, difficult near, difficult far).

In order to adequately normalize over subjects, the alpha modulation (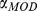) was calculated for each sensor which was defined as the ratio of the mean alpha power for covert visual spatial attention to a stimulus in the left hemifield (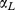) minus that for a stimulus in the right hemifield (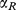), divided by the sum of both:
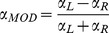
(1)


Additionally, we defined a region of interest (ROI) per hemisphere, which was determined as the set of synthetic planar sensors for which the alpha modulation averaged over all conditions was significantly different (p<0.05) from zero using a within-subject cluster-based nonparametric randomization test using 1000 randomizations [28], similar to the approach followed by Haegens et al. [29].

To combine the alpha modulation information from all sensors into one measure, we defined the alpha lateralization (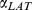) as the alpha modulation averaged over the sensors in the ROI in the left hemisphere (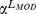) minus the alpha modulation averaged over the sensors in the ROI in the right hemisphere (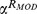):
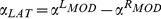
(2)


Finally, to compare the alpha lateralization between conditions we applied a two-way repeated measures ANOVA with factors Eccentricity (near, far) and Task Difficulty (easy, difficult) on the alpha lateralization.

#### Alpha time-power analysis

To investigate how the alpha power varied over time, we averaged the alpha power over all sensors in the ROI in the left hemisphere and over all sensors in the ROI in the right hemisphere for each time point and each trial. This resulted in a vector with the alpha power in the range between one second before cue onset until one second after target onset for each hemisphere (left, right) and trial. Preliminary investigations of the data revealed large variations in alpha power between subjects. To reduce this subject specific variance we normalized the data such that the average baseline power (−0.75 s to −0.25 s before cue onset) over both ROIs to zero mean and unit standard deviation over all trials. Next, we averaged over trials for each combination of subject, attended hemifield (left, right), condition (easy near, easy far, difficult near, difficult far), hemisphere (average left ROI, average right ROI), and time point. Trials were averaged relative to cue onset time. To explore in which combination of hemisphere, attended hemifield and time point differences in alpha power occurred between the two difficulty levels (easy and difficult), we averaged the previously calculated alpha power averages over each difficulty level (easy and difficult). To compare the alpha power over time between the easy and difficult conditions in the period between 1 s until 2.5 s after cue onset we applied a within-subject cluster-based nonparametric randomization test in each combination of hemisphere (average left ROI, average right ROI) and attended hemifield (left, right). Furthermore, we investigated the alpha lateralization over time for each difficulty level (easy, difficult). Therefore, we first calculated for each subject the alpha modulation (as defined in Equation (1)) for each combination of time, hemisphere (average left ROI, average right ROI) and difficulty level (easy, difficult). Next, we calculated the alpha lateralization as defined by Equation (2) for each difficulty level and time point. To evaluate when the alpha lateralization was deviating between the easy and difficult condition in the period between 1 s until 2.5 s after cue onset we used a within-subject cluster-based nonparametric randomization test with 1000 randomizations.

#### Classification analysis

Features for classification were obtained by averaging the log transformed alpha power for all sensors over the period from 1 s until 2.5 s after cue onset, resulting in a vector with 273 components for each trial. To provide the classifier only with data from sensors in the region where we expected the covert visual spatial attention signal, we selected sensors for each subject in a similar way as we did for the ROIs before (see Section Alpha power analysis), however now using a leave-subject-out procedure to prevent double dipping. Thus, we calculated for each subject two regions of interest using the data from all other subjects. For each subject the log alpha powers in the selected sensors were used as features for classifying left from right attention in each condition (easy near, easy far, difficult near, difficult far) using a linear support vector machine (SVM) classifier [30]. To test classification performance, ten-fold cross-validation was used, in which data is divided ten times in ten sequential folds, in which 90% of the data were used as training set and 10% of the data as test set. Average classification rates over folds were reported. An inner cross-validation was applied to optimize the regularization parameter of the classifier. To assess differences between conditions a two-way repeated measures ANOVA with factors Eccentricity (near, far) and Task Difficulty (easy, difficult) was applied on the classification accuracies.

## Results

### Behaviour

**Figure 2:**
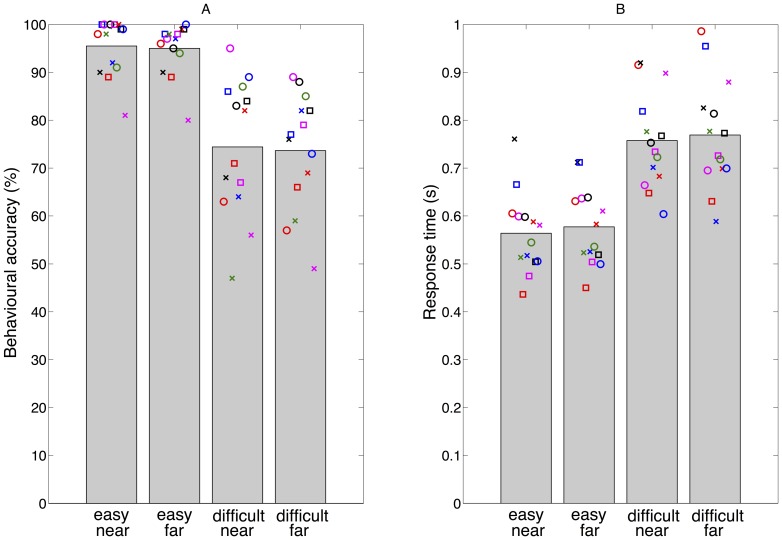
Behavioural accuracy (A) and response time (B) in the discrimination task. Bars show grand average behavioural accuracy as percentage correct (A) and grand average response time in seconds (B) within each condition (easy near, easy far, difficult near, difficult far). Markers indicate individual subject averages where each unique marker (combination of colour and shape) represents the same subject. On average subjects were less accurate with longer response times in the difficult conditions than in the easy conditions. No significant differences were found between behavioural accuracy or response time in the near and far spatial attention conditions for equal difficulty level.

Figure 2 shows the behavioural accuracy (Panel 2A) and response times (Panel 2B) in the orientation discrimination task. In line with our intentions, participants acquired on average an accuracy of 96% (near) and 95% (far) in the easy condition and an accuracy of 75% (near) and 76% (far) in the difficult condition. A two-way repeated measures ANOVA demonstrated a significant difference between easy and difficult conditions in accuracy (F(1,14) = 55.763, p<0.001). No effect of eccentricity was found (F(1,14) = 0.189, p = 0.671), indicating that we properly corrected for the size of the stimuli at the near and far eccentricities by taking the magnification factor into account. There was also no significant interaction effect between task difficulty and eccentricity (F(1,14) = 0.012, p = 0.915). As the accuracy distribution was significantly non-normal in the easy near condition (D(14) = 0.307, p<0.01), we also performed a non-parametric Wilcoxon signed-rank test on the behavioural accuracy between both levels of task difficulty. This test also showed a significant effect of task difficulty (p<0.001). Furthermore, as assessed using a binomial confidence interval for each subject the accuracy in the difficult condition deviated significantly from the accuracy in the easy condition (p<0.05).

For response times, a two-way repeated measures analysis also demonstrated a significant main effect of difficulty (F(1,14) = 88.805, p<0.001). On average the response time was 0.571 s in the easy condition and 0.763 s in the difficult condition. No effect of eccentricity was found (F(1,14) = 1.256, p = 0.283) and neither was an effect of interaction (F(1,14) = 0.015, p = 0.903).

Furthermore, a significant linear correlation was found between a subject's response time and accuracy (F(1,14) = 41.702, p<0.001, partial eta-squared = 0.762), indicating that the response time increased linearly with the accuracy of the response.

### MEG

**Figure 3:**
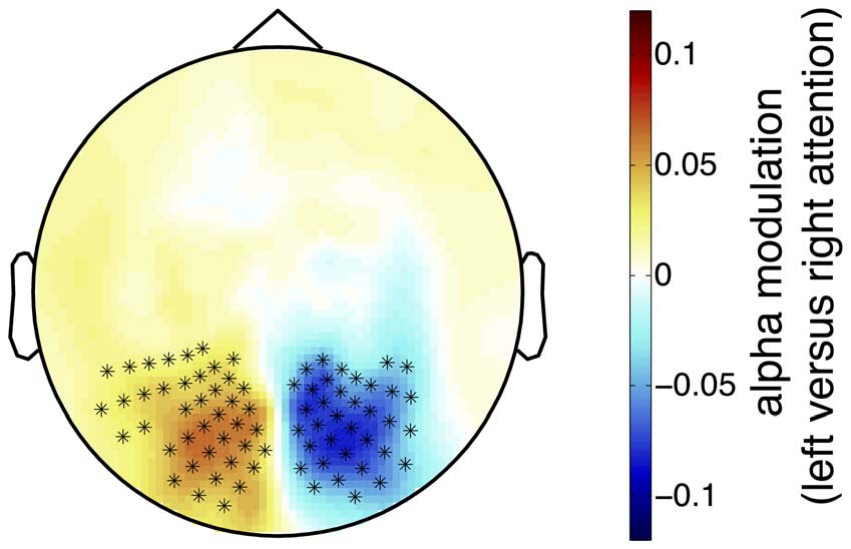
Grand average alpha modulation. Average alpha modulation over all conditions (easy near, easy far, difficult near, difficult far) and subjects (N = 14) in the period between 1 s and 2.5 s after cue onset. Crosses indicate sensors with an alpha modulation that deviate significantly from zero (p<0.05). These highlighted sensors define the ROIs (one in each hemisphere) used in the alpha power and alpha time-power analyses. Note that the small gap in the left ROI is due to sensor dropout.

For the MEG analyses, we determined for each hemisphere a ROI in which the alpha modulation, as defined by Equation (1), deviated significantly from zero over all conditions and subjects (see Figure 3). As expected, the sensors selected by this method are in the parietal and occipital cortex. The two hemispheric ROIs were used for the alpha power and alpha time-power analyses.

#### Alpha power analysis

**Figure 4:**
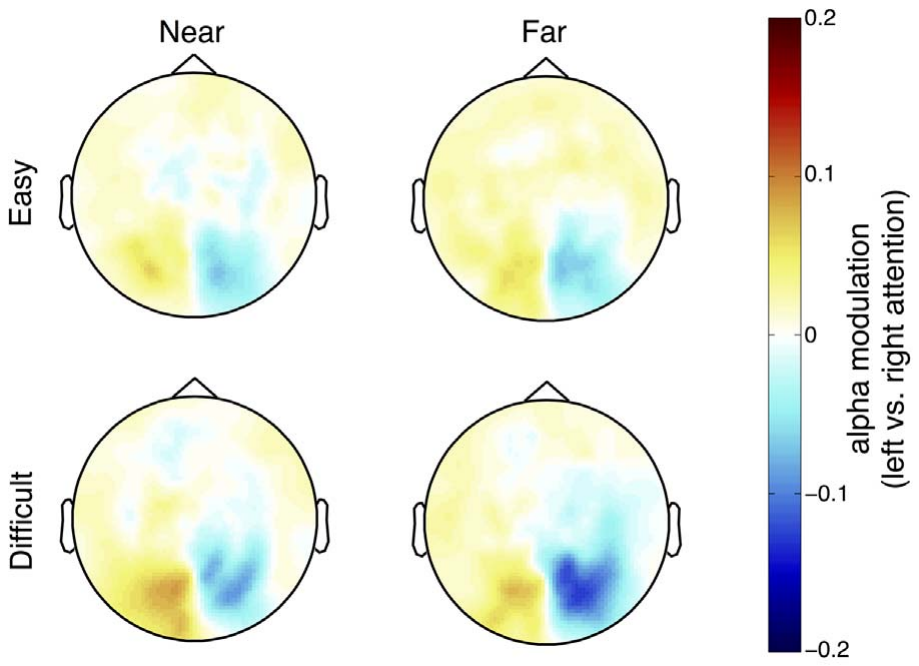
Grand average alpha modulation for each condition. Average alpha modulation as defined by Equation (1) over all subjects for each condition in the period between 1 s and 2.5 s after cue onset. The difficult conditions (bottom panels) show a more pronounced alpha lateralization than the easy conditions (p<0.01).

Figure 4 shows the alpha modulation as defined by Equation (1) averaged over all subjects for each condition (easy near, easy far, difficult near, difficult far) in the period between 1 s and 2.5 s after cue onset. To assess the effect of the different conditions, a two-way repeated measures ANOVA with Eccentricity and Task Difficulty was performed on the alpha lateralization, as defined by Equation (2). A significant main effect of task difficulty (F(1,14) = 13.437, p<0.01) was found. On average the alpha lateralization was 0.12 in the difficult condition compared to 0.08 in the easy condition. No effect of eccentricity was found (F(1,14) 2.593, p = 0.131) and neither was an interaction effect (F(1,14) = 0.401, p = 0.537). As the accuracy distribution was significantly non-normal in the difficult near condition (D(14) = 0.243, p<0.05), we also performed a Wilcoxon signed-rank test on the alpha lateralization between both levels of task difficulty. The result was significant (p<0.01).

#### Alpha time-power analysis

**Figure 5:**
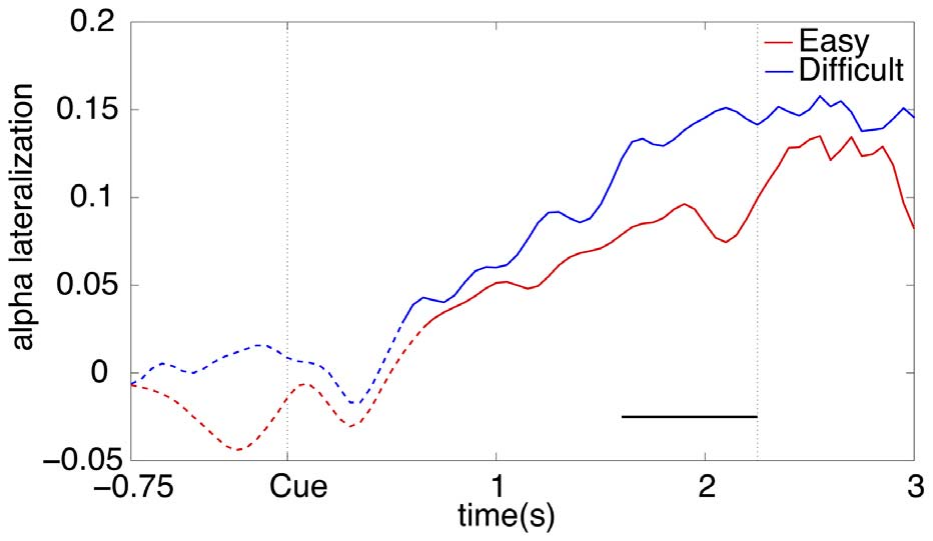
Grand average alpha lateralization over time for each difficulty level. The vertical dotted line at 2.25(p<0.01).

Next, we investigated the alpha lateralization over time for both difficulty levels (see Figure 5). Both in the easy and difficult condition the alpha lateralization is significantly different from zero from about 0.6 s after cue onset until target onset (p<0.01). A significant difference between task difficulty levels was found in the period between 1.6 s and 2.25 s after cue onset where the lateralization was more pronounced in the difficult condition than in the easy condition (p<0.01).

**Figure 6:**
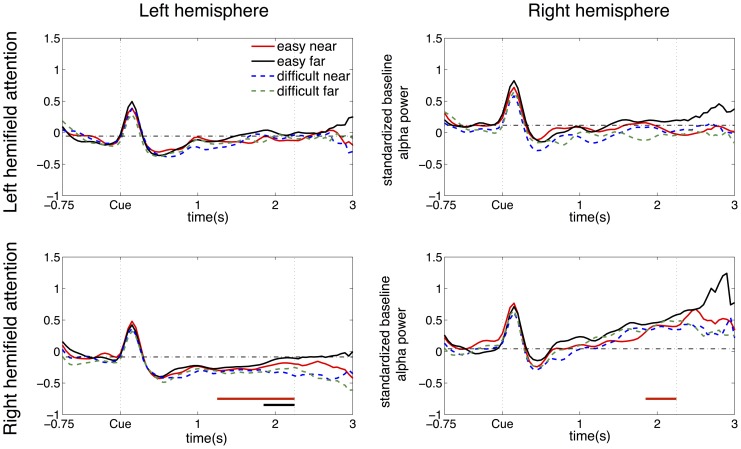
Grand average alpha power over time for each hemifield and hemisphere. Grand average alpha power over time for each combination of attended hemifield and hemisphere in the four conditions (easy near, easy far, difficult near, and difficult far) with attended hemifields (left and right) in the rows and hemispheres (left and right) in the columns. The horizontal dash-dotted line in each panel shows the baseline averaged over the four conditions in the time interval from −.75 s to −0.25 s before cue onset in that panel. The vertical dotted line at 2.25 s indicates the minimum trial length. Due to the variable trial length the standardized alpha power after 2.25 s is based on fewer trials. The red horizontal bars indicate a significant deviation from baseline (p<0.05). The black horizontal bar indicates a significantly lower alpha power in the difficult conditions compared to the easy conditions (p<0.05).

To further investigate from which hemisphere the differences in alpha lateralization might originate, we investigated the alpha power averaged over all subjects in each combination of attended hemifield and hemisphere. Figure 6 illustrates how the alpha power changes over time for each of the four conditions in each combination of attended hemifield and hemisphere. For each subject the data was standardized to the baseline as described in the method section. After a probably cue-related power increase directly after cue onset, a typical alpha decrease compared to baseline was shown contralateral to the attended hemifield (Figure 6, bottom-left and top-right panel), which was significant in the left hemisphere between 1.25 s and 2.25 s (p<0.05). An increase compared to baseline was shown ipsilateral to the right attended hemifield between 1.85 and 2.25 s (p<0.05) as shown in Figure 6, bottom-right panel. The alpha power tended to be lower for the difficult condition than for the easy condition in the left hemisphere when attending to the right hemifield (Figure 6, bottom-left). This effect was significant in the time interval between 1.85 s and 2.25 s after cue onset (p<0.05).

#### Classification analysis

**Figure 7:**
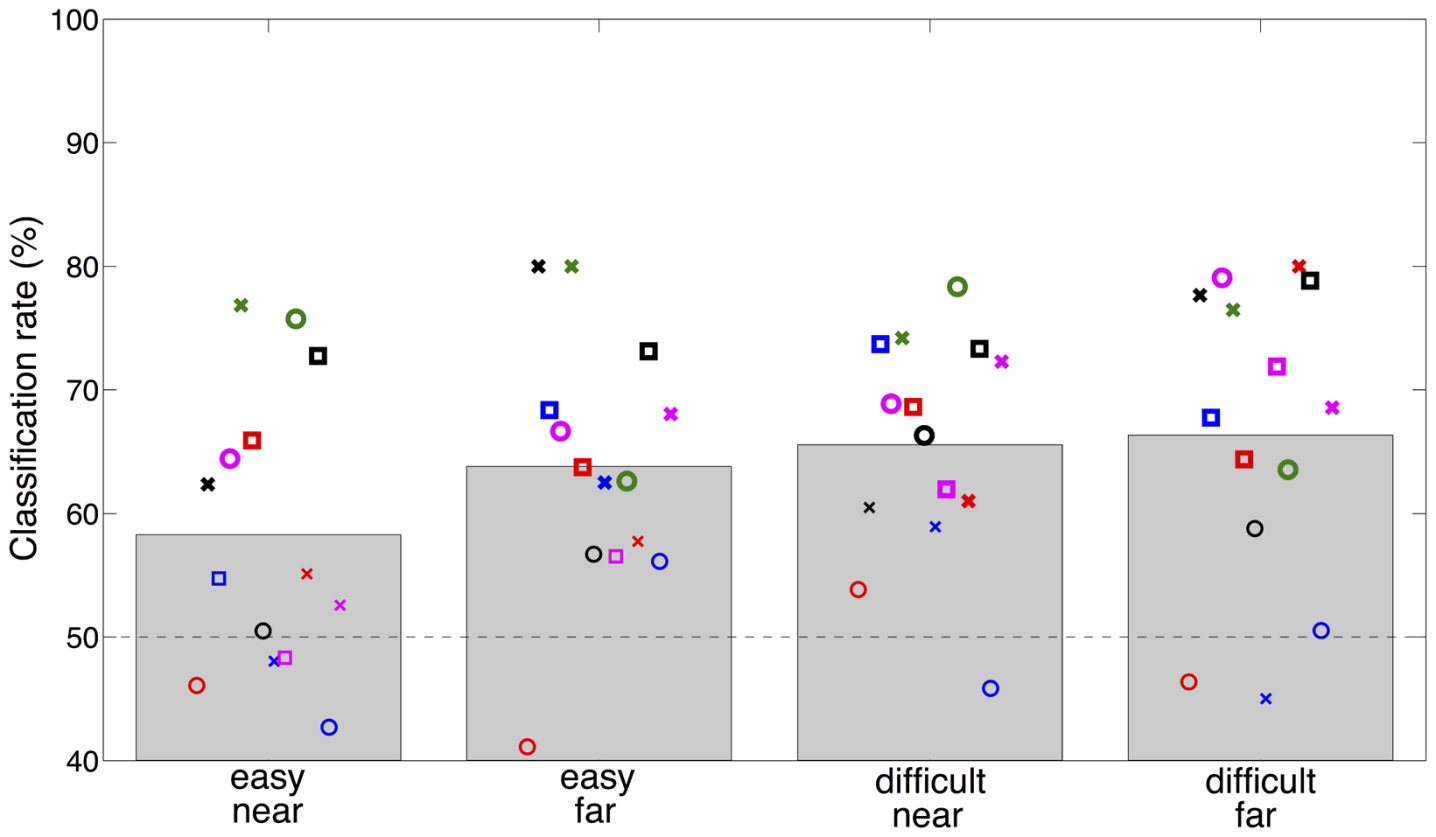
Classification rate of alpha power. Single-trial classification rate of alpha power in task-relevant sensors for each condition (easy near, easy far, difficult near, difficult far). Markers indicate individual subject classification rates whereby each unique marker (combination of colour and shape) represents the same subject. Bold markers indicate a classification performance significantly above chance level (p<0.05). On average a classification rate of 63.5% was reached (chance level is 50%). A significant difference was found between task difficulty levels; Classification rate was on average 61.1% in the easy condition and 65.9% in the difficult condition (p<0.05).

Figure 7 shows the mean classification rate and the classification rate for each subject for the four conditions (easy near, easy far, difficult near, difficult far). On average the classification rate was 63.5%. To evaluate the differences between the conditions a two-way repeated measures ANOVA with factors Eccentricity (near, far) and Task Difficulty (easy, difficult) was performed on the classification rates. In line with our expectations based on the grand average results, a significant effect of task difficulty was found (F(1,14) = 8.812, p<0.05). In the easy conditions the classification rate was on average 61.1% and in the difficult conditions the classification rate was on average 65.9%. Despite the visible difference in average classification rate between the easy near and easy far condition in Figure 7, no significant interaction effect was found (p = 0.17). A Pearson's correlation analysis between alpha lateralization and classification rate showed a strong positive correlation (r = 0.611, p<0.001), which suggests that the alpha lateralization was used for classification.

## Discussion

In this study we have examined the effects of task difficulty and eccentricity on the alpha lateralization (8 to 14 Hz) during covert visual spatial attention. Subjects performed a covert orientation discrimination task at an easy or difficult stimulus contrast at either a near (3.5°) or far (7°) eccentricity.

Behavioural analysis showed that task accuracy was lower and response times were slower in the difficult condition than in the easy condition, while no differences were found in task accuracy and response times between near and far spatial attention. This same pattern was found when investigating the alpha lateralization. The alpha lateralization was larger for a difficult task compared to an easy task, and was similar for attention to near and far spatial locations. The alpha lateralization difference between the easy and the difficult task was present in the period of 1.6 s until 2.25 s after cue onset, indicating a stronger lateralization before target onset in the difficult condition compared to the easy condition. Furthermore, a higher decrease in alpha power was found in the contralateral hemisphere during attention to the left hemifield in the difficult condition compared to the easy condition, indicating that to a large extent the alpha lateralization difference can be explained by the contralateral decrease of the alpha power. Classification rate of the alpha power in the brain regions involved during covert visual spatial attention was on average 63.5%. In the difficult conditions the classification rates were higher than in the easy conditions (65.9% and 61.1%, respectively).

### Task difficulty

Our results showed that a more difficult task led to an increase of the strength of alpha lateralization. This result obtained by MEG studies in humans is in line with a previous study using single-unit recordings in monkey visual cortex (area V1) on task difficulty, which also showed that task difficulty had an effect on neuronal processing. The latter study showed that an increased task difficulty enhanced neuronal firing rate at the focus of attention and suppressed it in the surrounding regions [19].

A contra-indication is a covert attention study in humans by Cosmelli et al. [31] in which no effect of task difficulty was found. In that study task difficulty was modulated to rule out modulations of alpha band activity due to non-specific changes such as alertness. Subjects were shown two coloured squares at the attended location and they had to press a button if one of these squares matched the colour they were instructed to detect. This resulted in a difference in behavioural performance for the different colours to be detected, as certain colours are easier to distinguish than others. However no differences in alpha power over the contralateral and ipsilateral parietal sites were found between the easy and difficult condition. In the study of Cosmelli et al. 50% of the targets were shown at the cued side, while in our study 100% of the targets were shown at the cued side. Previous research has shown that spatial certainty leads to graded changes in the extent to which alpha oscillations are lateralized [32]. A potentially weaker lateralization in the Cosmelli et al. study may mean that any additional modulation due to task difficultly is lost in the relatively stronger background noise.

Our results show that in both the easy and the difficult condition subjects show an alpha lateralization shortly after cue onset (see Figure 5) and the alpha lateralization increases more after cue onset for the difficult condition than for the easy condition. These results indicate that manipulating task difficulty has an effect, but do not address the actual cause of the change in alpha lateralization. For example, the effect could be due to a change in the visual task leading to differences in attentional demand or perceptual load or the participants could have changed their strategy during the difficult task by increasing their mental effort. Future work could alternatively investigate these causes in more detail. For instance, to independently manipulate mental effort a similar experiment could be conducted in which the maximum response time is manipulated, instead of the contrast of the target stimulus.

We discovered the largest changes in alpha power compared to baseline and effects of task difficulty in the left hemisphere while attending to the right hemifield and we assume that these changes also have affected the strength of the alpha lateralization. Our finding that the left hemisphere during right hemifield attention makes a large impact on the alpha lateralization is in agreement with previous research which showed that the left hemisphere mainly supports attention shifts to the right hemifield, while the right hemisphere is involved in shifts to both hemifields [33]. Quite remarkably, as we would expect the changes in alpha power to be symmetric with respect to attended hemifield we only found significant alpha power differences compared to baseline during right hemifield attention. Recently, this bias to the right visual hemifield has also been found in ADHD patients [34].

A possible caveat in our experimental design was that attention to the cued location in the easy condition might not have been necessary as the targets in the easy condition were presented at maximal contrast. Subjects could have detected the orientation of the contrast due to a pop-up effect of the target. However, we found a significant alpha lateralization pattern in the easy condition indicating that our participants did pay covert attention to the visual stimuli in the easy condition. This lateralization pattern became significantly different from zero shortly after cue onset, as was also the case in the difficult condition.

Previous covert visual attention studies reported correlations between pre-target alpha modulation and behavioural measures as summarized in [8]. We performed similar correlation analyses as used in [35], [36], but did not find a significant correlation between the alpha modulation and either the behavioural accuracy or response times during the difficult condition. Furthermore, we have analysed the microsaccade rate between the easy and difficult condition using the algorithm proposed by [37]. Microsaccade rate decreased before target onset in both conditions indicating focused attention [38]. Additionally, the microsaccade rate was slightly, but significantly higher in the difficult condition than in the easy condition in the period after cue onset (0–1.8 seconds) but this effect disappeared near target onset.

### Eccentricity

Contrary to our expectations, we did not find an effect of eccentricity with our alpha lateralization analyses. Bahramisharif et al. [14] did find different alpha lateralization patterns for different eccentricities. We believe that the most plausible explanation for the difference with the results in our study is that we corrected for potential confounds of cortical magnification [16] and task difficulty, which were not taken into account by Bahramisharif et al. [14].

One could argue that we did not observe an effect of eccentricity, because we averaged over the ROIs such that any changes in location within the ROIs may have been obscured. However, a post-hoc within-subject cluster randomization test [28] to identify clusters of sensors in which the alpha lateralization varied between near and far conditions out of all sensors also did not reveal any significant clusters.

### BCI performance

Based on the stronger alpha modulation in the difficult versus the easy condition, we expected to see a higher classification rate in the difficult conditions compared to the easy conditions. This higher classification rate was indeed found. This effect was found when using the alpha power of sensors from the ROIs as features. We chose to report these ROI sensor results, as these features gave on average the highest classification performance. However, we also observed the task difficulty effect when using the alpha power from all sensors as separate features for classification, indicating that the selection of sensors did not alter the observed effects. Furthermore, Figure 7 seemed to indicate that the classification rate in the easy far condition was more similar to the difficult condition than to the easy near condition, although there was no significant interaction effect. When using all the sensors as separate features for classification, the easy far condition was more similar to the easy near condition with classification rates of respectively 60.4% and 59.4%, while the difficult far and difficult near conditions had classification rates of respectively 64.7% and 65.3%. This result, using a different subset of sensors, indicates as well that the higher classification rate in the easy far condition in Figure 7 is not a robust effect. The positive correlation of 0.611 between the classification rate and the alpha lateralization showed that the classification analysis was for a large part using similar information as the lateralization analysis. While this was an MEG study and therefore not directly applicable for BCI, we expect the results to transfer to EEG since the visual covert attention paradigm seems to give similar brain signals in EEG [13]. Although the recorded brain signals will be more spread over the scalp due to increased spatial smearing in EEG, we expect the effect of difficulty to remain as it is an effect in the magnitude of the alpha power. Average classification rates in Treder's et al. [13] EEG study were higher (74.6%) than in our MEG study (63.5%), however in their study performance was improved by selecting the subject-specific pair of directions which maximised classification performance.

### Conclusion

Surprisingly, we did not find an effect of target eccentricity on the alpha lateralization during the covert visual spatial attention task. Task difficulty did show an effect on the alpha lateralization. A more difficult task increased the alpha lateralization and the BCI classification rate. This implies that it is important to consider the influence of task difficulty in alpha oscillation studies and from a BCI perspective it seems important to have a difficult task in a covert visual spatial attention paradigm in order to maximize performance.
